# Epigenetic modifications of tumor necrosis factor-alpha in joint cartilage tissue from osteoarthritis patients - CONSORT

**DOI:** 10.1097/MD.0000000000027868

**Published:** 2021-12-23

**Authors:** Qiang Zhang, Zhengxiao Ouyang, Xiaoxia song, Wei Zhu, Xinqiao Tang, Zhong Liu, Xiaoming Chen

**Affiliations:** a Department of Orthopedics, the Central Hospital of Xiangtan City, Xiangtan, Hunan, P.R. China; b Department of Orthopaedic, the Second Xiangya Hospital, Central South University, Changsha, Hunan, China; c Department of Respiratory Medicine, the Central Hospital of Xiangtan City, Xiangtan, Hunan, P.R. China; d Department of Orthopedics, the Central Hospital of Changsha City, Changsha, Hunan, P.R. China.

**Keywords:** epigenetic modifications, joint cartilage, osteoarthritis, tumor necrosis factor-alpha

## Abstract

**Background:**

Osteoarthritis (OA) remains one of the most common osteopathy for centuries, which can be attributed to multiple risk factors including mechanical and biochemical ones. More and more studies verified that inflammatory cytokines play important roles in the progression of OA, such as tumor necrosis factor-alpha (TNF-α). In this study, we aimed to investigate the relationship between epigenetic manifestations of TNF-? and the pathogenesis of OA.

**Methods:**

Totally, 37 OA patients’ cartilage was collected through the knee joint and 13 samples of articular cartilage as healthy control was collected through traumatic amputation. Real-time PCR, Western blot and ELISA analysis were performed to observe the expression of target genes and proteins in collected samples.

**Results:**

Compared with the healthy control group, TNF-? was over-expressing in cartilage which was collected from OA patients. DNA hypomethylation, histone hyperacetylation and histone methylation were observed in the TNF-? promoter in OA compared with normal patients, and we also studied series of enzymes associated with epigenetics. The results showed that by increasing DNA methylation and decreasing histone acetylation in the TNF-? promoter, and TNF-? over-expression in OA cartilage was suppressed, histone methylation has no significant correlation with OA.

**Conclusion:**

In conclusion, the changes of epigenetic status regulate TNF-α expression in the cells, which are pivotal to the OA disease process. These results may give us a better understanding of OA and may provide new therapeutic options.

## 1 Introduction

Osteoarthritis (OA) is the most common degenerative disease of the synovial joint among the elderly population,^[1]^ caused by degeneration of articular cartilage. At present, the specific cause of OA is unclear.^[2]^ More and more attention have been paid to the role of cytokines in OA.^[3–5]^ Studies have confirmed that OA is the result of the imbalance between the anti-inflammatory factors and pro-inflammatory factors,^[6]^ which destroys their stability. These cytokines and growth factors are secreted by chondrocytes and synovial cells, including matrix metalloproteinases and tissue inhibitors of metalloproteinases family, interleukin family (IL), tumor necrosis factor family (TNF) and vascular endothelial growth factor family, etc.

Numbers of mechanisms can regulate gene expression and cell fate persistence,^[7–10]^ commonly referred to as epigenetics, while existing different forms of performance, such as histone modifications, microRNAs and long non-coding RNAs, the most studies in OA is decidedly DNA methylation.^[3]^ DNA methylation is the methylation of 5’—C—phosphate—G—3’ (CpG) residues to cytosine nucleotides, Whether CpG methylation depends on local gene sequences.^[11]^ There are many studies on the relationship between epigenetics and osteoarthritis, DNA methylation can modulate OA genetic susceptibility loci, with altered methylation impacting the effect of the OA-associated single-nucleotide polymorphism (SNP) rs143383 on GDF5 expression.^[12]^ CpG methylation can regulate allelic expression of GDF5 by modulating the binding of SP1 and SP3 repressor proteins to the osteoarthritis susceptibility SNP rs143383.^[13]^

As a key inflammatory cytokine, TNF-α involves in the pathophysiological processes occurring in the course of OA.^[5]^ Results showed that^[14]^ TNF-α could induce MMPS, PGE2, NO, then reduce the production of collagen and proteoglycan and promote the degradation, the decrease of the important components of the two kinds of chondrocytes, which leads to the reduction of chondrocytes and the destruction of articular cartilage. In addition, TNF-α can induce apoptosis in sensitive cells by the receptor on the target cell membrane. In recent years, more and more studies have shown that the change of gene expression level may affect the development of some diseases. Genomic DNA methylation, as a form of epigenetics, plays a key role in some diseases. It has been proved that the methylation status of TNF- plays an important role in liver failure,^[15]^ heart failure^[16]^ and lipolysis.^[17]^

Epigenetic modifications are reversible and heritable, which includes changes in DNA methylation patterns, modification of histones and altered microRNA expression levels.^[18]^ A report showed that the epigenetic modifications in IL-6 promoter in synovial fibroblasts were involved in the pathophysiology of OA.^[19]^ Meanwhile, there is no report on the level of epigenetic modifications of promoter region of TNF-α gene in the cartilage of OA. Therefore, in the present study, we attempted to investigate it. Cartilage was isolated from OA patients. DNA methylation, histone acetylation and histone methylation were investigated. We also studied a series of enzymes associated with epigenetics: methyl-CpG binding protein 2, DNA (cytosine-5-)-methyltransferase 1,3a, HDAC1, histone acetyltransferase 1, CREB binding protein and p300. Further, by altering the status of DNA methylation and histone acetylation in the TNF-α promoter, TNF-α over-expression was suppressed in the cartilage region of OA.

To sum up, as one of the most common degenerative diseases among the elderly population, OA was previously confirmed as the result of inflammatory factors imbalance. As a key inflammatory cytokine, TNF-α is reported to be involved in the pathophysiological processes in OA since it has been shown to reduce the production of collagen and proteoglycan and promote the degradation, thus leading to the destruction of articular cartilage. However, no evidence on the level of epigenetic modifications of promoter region of TNF-α gene in the cartilage of OA has been reported. Therefore, in this study, we hypothesized that in the pathogenesis of OA, DNA methylation and histone acetylation in the TNF-α promoter could be dysregulated. And by studying OA samples and in vitro cell models, we aimed to investigate the relationship between epigenetic manifestations of TNF-α and the pathogenesis of OA.

## 2 Material and methods

### 2.1 Patients and sampling

The Human Research Ethics Committees of Xiangya No.2 Hospital of Central South University has approved this research and all methods were performed following the last vision of the Declaration of Helsinki. Written informed consent was obtained from all patients who were recruited between June 2016 and March 2018. Human cartilage was isolated through the knee joint which from 37 OA patients receiving knee replacement surgery and 13 samples of articular cartilage as healthy control was collected through traumatic amputation, According to the diagnostic standard of osteoarthritis in China in 2007, excluding (1) secondary osteoarthritis (2) iatrogenic factors; (3) osteoarthritis with other serious diseases. Cartilage cells were isolated cells and filtered through 70-μm nylon filters. The cells were plated on cell culture dishes in 5% CO_2_ in DMEM (Life Technologies, Grand Island, NY, USA) supplemented with 10% fetal bovine serum (FBS), 100 U/ml penicillin, and 100 μg/ml streptomycin. Fibroblasts between passages 3 to 5 were used for the experiments.

### 2.2 Real-time PCR

Total RNA was harvested from cartilage using the RNeasy Mini Kit (Qiagen, Valencia, CA). For RT-PCR, cDNA was reverse-transcribed from 1 μg of total RNA. Quantitative PCR was performed using SYBR Green PCR Master Mix (Takara Bio Inc., Otsu, Japan) on ABI Prism 7500 PCR machine (Applied BioSystems, Foster City, CA). The following cycling conditions were used: 94 °C for 30 seconds, followed by 40 cycles of 94 °C for 5 seconds and 60 °C for 34 seconds. The semi-quantitative 2 − ΔΔCt method was employed to calculate the relative expression level of the target gene. β-actin was used as the loading control. Primer sequences:

TNF-α: 5’-GGTTTAGAAGATTTTTTTCGGAATC-3’ (forward), 5′- TAAACCCTACACCTTCTATCTCGAT -3′ (reverse);
Dnmt1: 5’-CCTAGCCCCAGGATTACAAGG-3’ (forward), 5′- ACTCATCCGATTTGGCTCTTTC-3′ (reverse);
Dnmt3a: 5’-CACCGGCCATACGGTGG-3’ (forward), 5′-CAGCAGCCATTTTCCACTGC-3′ (reverse);
HDAC1: 5’-CGCCCTCACAAAGCCAATG-3′ (forward), 5′-CTGCTTGCTGTACTCCGACA-3′ (reverse);
HAT1: 5’-TACAGCGGAAGATCCATCCAA-3’ (forward), 5′-CTGTTGTGCCTCTATCGCCA-3′ (reverse);
GAPDH: 5’-GGAGCGAGATCCCTCCAAAAT-3’ (forward), 5′-GGCTGTTGTCATACTTCTCATGG-3′ (reverse).

### 2.3 Western blot

Cells were lysed on ice for 30 min in a buffer containing 50 mM Tris-HCl, pH 7.4, 150 mM NaCl, 1% Nonidet P-40, and 0.1% SDS supplemented with protease inhibitors (10 g/ml leupeptin, 10 g/ml pepstatin A, and 10 g/ml aprotinin). The proteins were separated by SDS-PAGE, transferred to a nitrocellulose membrane, and detected using anti- TNF-α(#12912, Cell Signaling Technology, Danvers, MA), anti-HA tag (#3724, CST), and anti-β-actin (#3700, CST) antibodies. The proteins were visualized using an enhanced chemiluminescence system (GE Healthcare, Piscataway, NJ).

### 2.4 ELISA

Normal and OA cartilage were cultured in a complete medium for 24 h, then the complete medium was replaced by a serum-free medium. After 2 h, the conditioned medium was harvested, centrifuged to remove particulate matter, and stored at −20 °C until analyzed. Quantitation of TNF-α secreted into the medium was determined using a quantitative sandwich ELISA for human TNF-α according to the manufacturer's instructions (R&D Systems, Minneapolis, MN).

### 2.5 DNA isolation and bisulfite sequencing PCR

Genomic DNA of human cartilage (5 mg) was denatured by 2 M NaOH for 15 min at 50°C. 2% low-melting agarose were subsequently added to the DNA solution, and agarose beads were formed after pipetting 15-μl DNA/agarose mixture into cold mineral oil. The DNA/agarose beads were treated with freshly prepared hydroxyquinone (10 mM; Sigma) and sodium bisulfite (40.5%, pH 5; Sigma) at 50°C for 16 h under mineral oil. The reaction was stopped by 0.3 M NaOH for 10 min at room temperature.

The PCR amplifications were performed in 25 μl reactions containing one agarose/DNA bead. The following primer sequences were used: 5′-ATTAATATTATAGATAATT-3′ (forward), 5′-ATTATATATTTATTATTGTGT-3′ (reverse); The PCR products were separated by agarose gel, purified and cloned into the pMD18-T vector system (Takara). Fifteen clones of each sample were sequenced, and the sense strands were used to evaluate CpG site methylation status.

### 2.6 Chromatin immunoprecipitation (ChIP)

The cells were cross-linked with 1% formaldehyde for 10 min at 37 °C, and crude nuclei were recovered. The crude nuclei were sonicated to produce 500 bp chromatin fragments. The following antibodies were used in the ChIP assay: MeCP2, H3K9 di-methylation, HDAC1, Dnmt1, Dnmt3a, SETDB1, MLL2 (Abcam), H3K9/K14 acetylation, H4K12 acetylation, H3K4 tri-methylation, H3K27 tri-methylation (Upstate), HAT1 (GeneTex), CBP (Abcam), p300 (Abcam) and Ezh2 (Cell Signaling Technology, Danvers, MA, USA). IgG (Sigma) was used as a negative control. For each ChIP assay, 5 μg antibodies were added, and the samples were incubated overnight at 4 °C. The ChIP and input DNA samples were quantified using quantitative PCR. Primer sequences used in ChIP-qPCR: 5’-ATTAAATCCCGATAGTATACC-3’ (forward), 5’-ATCATAGTTAGCAATTCAATGCAGTAT-3’ (reverse);

### 2.7 In vitro methylation and luciferase reporter assay

M.SssI (New England Biolabs, Ipswich, MA) was employed to in vitro methylate CpG sites for 6 hours at 37°C. The treated DNA fragments and vectors were ligated and subsequently purified using phenol/chloroform extraction and ethanol precipitation.

Cells were plated onto 24-well plates. Transient transfections were performed using Fugene6 (Roche); the phRL-SV40 vector (Promega, Madison, WI) was used as the control for transfection efficiency. Forty-eight hours after transfection, both firefly and Renilla luciferase activities were measured using a dual-luciferase reporter assay system (Promega). The relative luciferase units (RLUs), the ratios of firefly to Renilla luciferase activities, were subsequently calculated.

### 2.8 Lentivirus

Lentiviral vectors containing the coding sequences of Dnmt3a were purchased from Genecopoeia (Rockville, MD). 293T cells were plated onto a 10-cm dish, and the transfection mixture was added directly to the culture medium at 70% to 80% confluence. Following transfection, the samples were incubated in a CO_2_ incubator at 37°C for 48 h and the culture medium containing virus particle was subsequently collected.

### 2.9 Statistical analysis

As for the statistical analysis of the data from human samples that followed normal distribution, Student *t*-test was used for two-group comparisons and one-way ANOVA was used for multiple comparisons. Tukey's test was employed to evaluate significant differences using ANOVA. For those data that did not follow normal distribution, the nonparametric method was employed unless data scaling and transformation were successful. https://www.qualtrics.com/blog/calculating-sample-size/ was used to evaluate the sample size needed for the study. As for the statistical analysis for the in vitro data, Student *t*-test was used for two-group comparisons and one-way ANOVA was used for multiple comparisons. Tukey's test was employed to evaluate significant differences using ANOVA. All experiments were repeated at least three times. Overall, *P* values < .05 were regarded as significant. All data are presented as the means ± SD unless otherwise specified.

## 3 Results

### 3.1 TNF-α is up-regulated in OA cartilage

**Figure 1:**
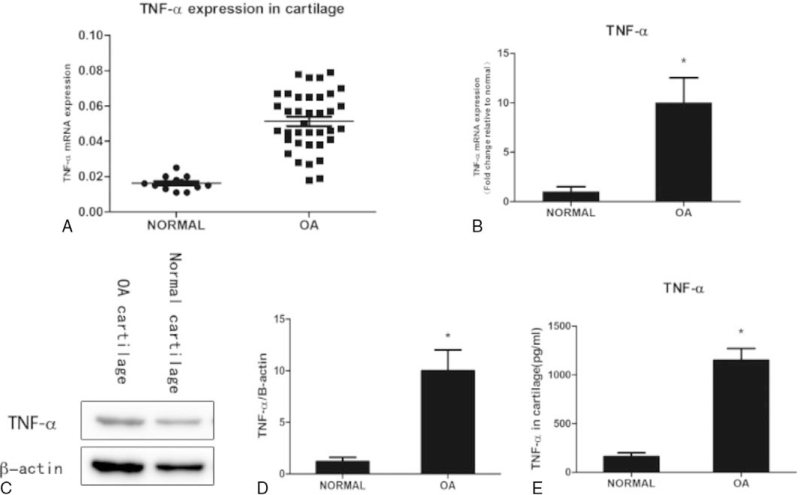
TNF-α is up-regulated in OA cartilage. Figure 1: TNF-α expression in cartilage (A). Cartilage was harvested from patients with upper limb amputation due to trauma and without osteoarthritis were diagnosed and OA patients and cultured in vitro. TNF-α expression in cartilage samples from normal (n = 13) and OA knee (n = 37) patients was assayed by qRT-PCR and normalized to 18 s. Bars represent the mean. ∗*P* < .05, Mann–Whitney *U* test. Total RNA was isolated for quantitative RT-PCR using TNF-α primers (B), Used β-actin as internal control. Total protein was harvested for western blot analysis using TNF-α antibody (C, D) β-actin was used as an internal control. The relative protein expression level of TNF-α was normalized to β-actin. The TNF-α level was measured in cartilage from non-arthritic donors and OA patients using ELISA (E). Data are shown as the means ± SD. ∗*P* < .05, OA Cartilage vs. normal Cartilage, the data were obtained from 37 OA patients receiving knee replacement surgery and 13 samples of non-arthritic tissues obtained during amputation.

Cartilage was harvested from patients who suffered from car accidents or other trauma in the middle and lower limb amputation at Xiangya No.2 Hospital of Central South University during 2016.6∼2017.3 and OA patients and cultured in vitro. Total RNA was isolated for quantitative RT-PCR using TNF-α primers. The results showed the TNF-α mRNA was overexpressed in OA cartilage compared with normal controls. The significant upregulation of TNF-α protein was observed in OA cartilage compared with normal controls. The results revealed higher TNF-α levels in cartilage from OA patients compared with normal controls (Fig. 1).

### 3.2 TNF-α is hypo-methylation in OA cartilage

**Figure 2:**
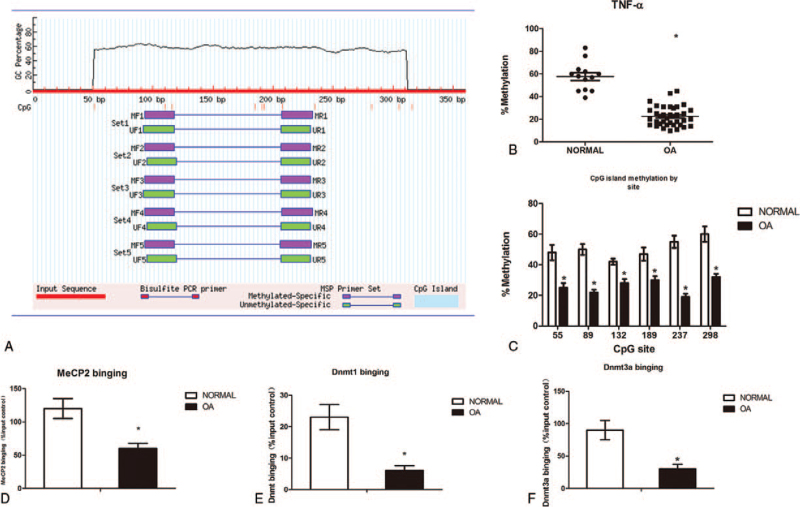
DNA hypo-methylation in the TNF-α promoter in cartilage from OA patients. Figure 2: Methylation of the TNF-α locus in non-arthritic OA knee cartilage. Methylation was assessed by pyrosequencing of bisulphite converted genomic DNA. The CpG island spans from 50 to 320 bp upstream of the TNF-α TSS,which contains lots of CpG sites(A). Percentage overall methylation of the CpG island in cartilage from Normal (n = 13) and OA knee (n = 37) patients(B). Methylation of the TNF-α island by CpG site(C). ChIP was performed to measure MeCP2 (D), Dnmt1 (E) and Dnmt3a (F) binding to the TNF-α promoter in normal and OA cartilage. IgG was used as a negative control. The results are expressed as the percentage of MeCP2 and Dnmt1/3a binding in the input control. Data are shown as the means ± SD. ∗*P* < .05, OA cartilage *vs.* normal cartilage. The data were obtained from 37 OA patients receiving knee replacement surgery and 13 samples of non-arthritic tissues obtained during amputation.

TNF-α promoter contains abundant CpG sites (i.e., potential DNA methylation targets) around the transcription start site (TSS), The sequence of TNF-α gene was found in GenBank, and the promoter region of the CpG gene was located in the CpG software: MethPrimer computer program (http://www.urogene.org/methprimer/). The CpG island is located between 50 and 320 bp upstream of the TNF-α TSS (Fig. 2A), and was significantly hypo-methylation in OA knee cartilage (n = 37, *P* < .05) relative to non-OA cartilage (n = 13). The methylation status of these CpG sites was investigated in normal and OA cartilage. Significant DNA hypo-methylation was observed in OA cartilage compared with normal controls (Fig. 2B and C). As in the TNF-α promoter, the Dnmt family is responsible for de novo adding a methyl group to cytosine, while MeCP2 specifically binds to the DNA methylation sites, So, we performed ChIP assays to measure MeCP2, Dnmt1 and Dnmt3a binding. The results revealed weaker bindings in the TNF-α promoter in OA cartilage compared with normal controls (Fig. 2D, E and F).

### 3.3 Inhibition of DNA methylation in articular chondrocytes results in the increased expression of TNF-α promoter

**Figure 3:**
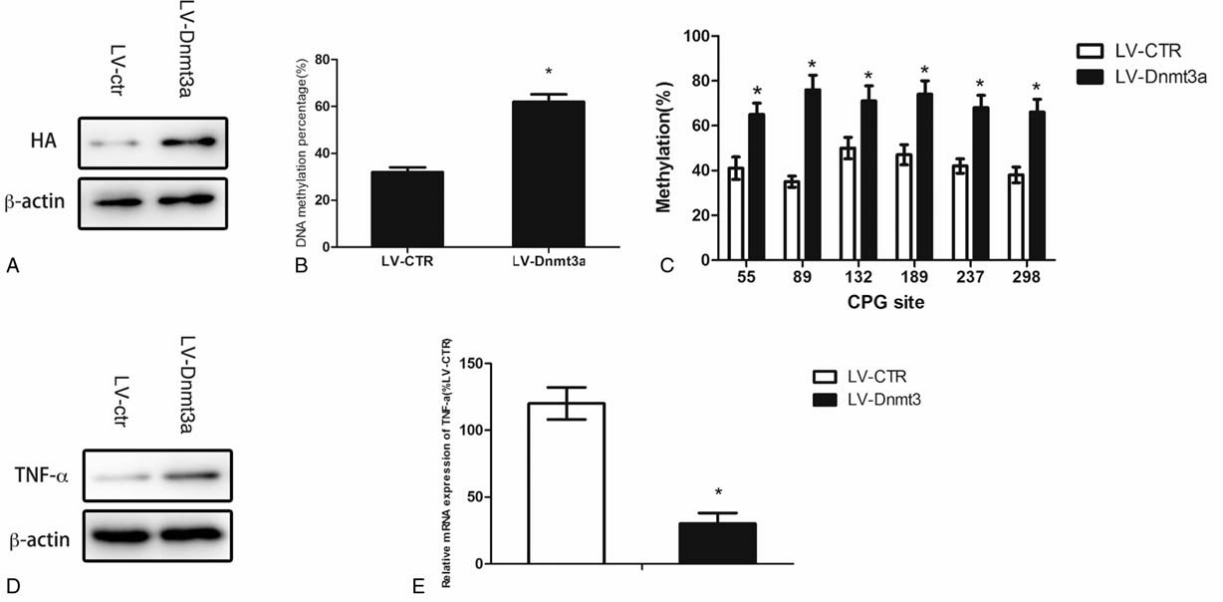
Inhibition of DNA methylation in articular chondrocytes results in the increased expression of TNF-α promoter. Figure 3: OA cartilage was infected with lentivirus loaded with HA-tagged Dnmt3a (A) Empty vector was used as a control. Genomic DNA was isolated from OA cartilage with Dnmt3a over-expression. DNA methylation percentage was calculated, and the results were expressed as the percentage of methylated CpG sites in all of CpG sites (B). The DNA methylation statuses in the TNF-α promoter were investigated (C). Total protein was isolated for western blot analysis using TNF-α antibody (D) ,β-actin was used as an internal control. Total RNA was isolated from OA cartilage with and without Dnmt3a over-expression for quantitative RT-PCR using TNF-α primers (E). Data are shown as the means ± SD. ∗*P* < .05, LV-Dnmt3a vs. LV-ctr. The data were obtained from 37 OA patients receiving knee replacement surgery and 13 samples of non-arthritic tissues obtained during amputation.

We next verdict with the alterations of the status of DNA methylation, TNF-α over-expression in OA cartilage could be modulated. Lentivirus loaded with HA-Tagged Dnmt3a was used to infect articular cartilage (Fig. 3A). We found that with the Dnmt3a over-expression, the TNF-α promoter was methylated (Fig. 3B and C). Total RNA and protein were harvested from OA cartilage with and without Dnmt3a over-expression for quantitative RT-PCR and western blot analysis to measure TNF-α expression levels. The results revealed that both the mRNA (Fig. 3D) and protein (Fig. 3E) levels of TNF-α in OA cartilage could be significantly suppressed after elevating the DNA methylation of the TNF-α promoter. The results revealed the significantly lower levels of TNF-α secretion from OA cartilage with Dnmt3a overexpression, compared to without overexpression.

### 3.4 Histone hyper-acetylation in the TNF-α promoter in cartilage from OA patients

**Figure 4:**
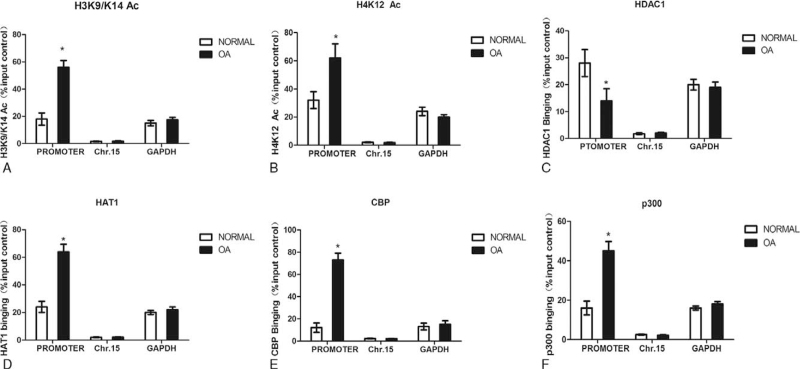
Histone hyper-acetylation in the TNF-α promoter in cartilage from OA patients. Figure 4: ChIP assays were used to examine acetylated H3K9/K14 (A), H4K12 (B), HDAC1 (C), HAT1 (D), CBP (E) and p300 (F) occupancy in the region of the TNF-α promoter in normal and OA cartilage. Chromosome 15 and GAPDH were used as controls in the ChIP-qPCR experiments. The results were normalized to the percentage in the input control. Data are shown as the means ± SD. ∗*P* < .05, OA vs. normal. The data were obtained from 37 OA patients receiving knee replacement surgery and 13 samples of non-arthritic tissues obtained during amputation.

There may be a large number of acetylation sites in the tail of histone H3/H4, which may be involved in gene transcription. So we also studied TNF-α promoter histone modifications in normal and OA cartilage. In our study, ChIP assays were used to examine the histone tails acetylation in the TNF-α promoter in normal and OA cartilage; H3K9/K14 and H4K12 acetylation were selected to represent the acetylation of H3 and H4, Using IgG as negative control. Representative silent (Chromosome 15) and active (GAPDH) chromatin regions as controls in the ChIP-qPCR experiments. The result showed significant increases in H3K9/K14 (Fig. 4A) and H4K12 (Fig. 4B) acetylation within the TNF-α promoter in OA cartilage compared with normal controls. We also found that the GAPDH promoter has more acetylated histone H3 and H4 occupancy than the silent region on Chromosome 15, which correlates positively with gene activity. HDAC1, HAT1, CBP and p300 binding, as for removing and adding the acetyl groups, we also used ChIP assays to measure it. The results showed weaker HDAC1 binding (Fig. 4C) and stronger HAT1 (Fig. 4D) and CBP binding (Fig. 4E) in the TNF-α promoter in OA cartilage compared with normal controls. Meanwhile, stronger p300 binding was observed in the TNF-α promoter in OA cartilage compared with normal controls (Fig. 4F).

### 3.5 Inhibition of Histone acetylation in articular chondrocytes results in the increased expression of TNF-α promoter

**Figure 5:**
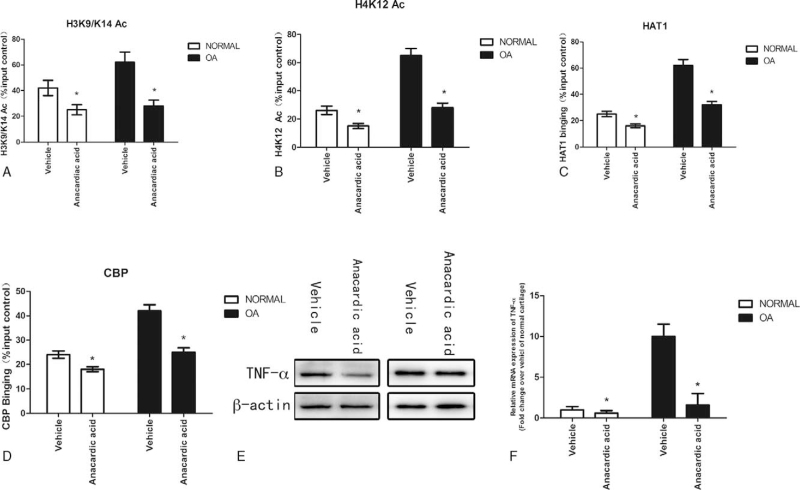
Inhibition of Histone acetylation in articular chondrocytes results in the increased expression of TNF-α promoter. Figure 5: Normal and OA cartilage was treated with anacardic acid (4μM). ChIP was performed to measure acetylated H3K9/K14 (A), acetylated H4K12 (B), HAT1 (C) and CBP (D) occupancy in the region of the TNF-α promoter in normal and OA cartilage treated with anacardic acid. The results were normalized to the percentage of the input control. Total protein was isolated for western blot analysis using TNF-α antibody (E) β-actin was used as an internal control. Total RNA was isolated from normal and OA cartilage with and without anacardic acid treatment for quantitative RT-PCR using TNF-α primers (F). Data are shown as the means ± SD. ∗*P* < .05, ∗*P* < .05, anacardic vs vehicle. The data were obtained from 37 OA patients receiving knee replacement surgery and 13 samples of non-arthritic tissues obtained during amputation.

We employed anacardic acid to treat normal and OA cartilage. H3K9/K14 (Fig. 5A) and H4K12 (Fig. 5B) acetylation was inhibited in response to anacardic acid treatment. Accordingly, the binding of HAT1 (Fig. 5C) and CBP (Fig. 5D) on the TNF-α promoter was also inhibited in response to anacardiac acid treatment. Through quantitative RT-PCR and western blot, the results showed that both the mRNA (Fig. 5E) and protein (Fig. 5F) levels of TNF-α in normal and OA cartilage significantly suppressed after decreasing the histone acetylation of the TNF-α promoter. The results revealed the significantly lower levels of TNF-α secretion from normal and OA cartilage with anacardic acid treatment, compared with vehicle treatment.

### 3.6 Histone methylation in the TNF-α promoter in cartilage from OA patients

**Figure 6:**
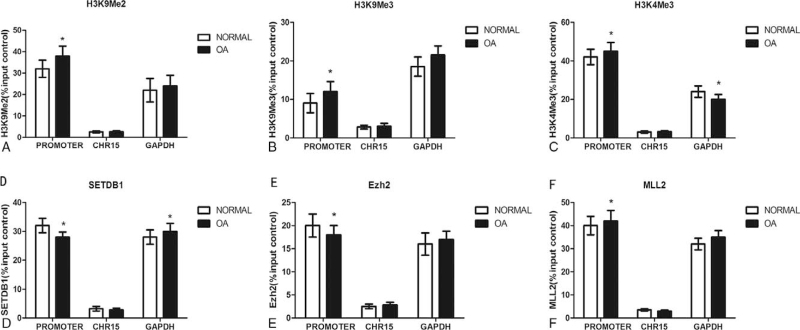
Histone methylation in the TNF-α promoter in cartilage from OA patients. Figure 6: ChIP assays were used to examine di-methylated H3K9 (A), tri-methylated H3K27 (B), tri-methylated H3K4 (C), SETDB1 (D), Ezh2 (E) and MLL2 (F) occupancy in the region of the TNF-α promoter in normal and OA cartilage. The results were normalized to the percentage in the input control. Data are shown as the means ± SD. The data were obtained from 37 OA patients receiving knee replacement surgery and 13 samples of non-arthritic tissues obtained during amputation.

As we know, the location and degree of Histone methylation can lead to either gene activation or repression. In this study, we used ChIP assays to examine H3K9 di-methylation, H3K27 tri-methylation (repressive markers) and H3K4 tri-methylation(active marker) in the TNF- promoter in normal and OA patients. There is no statistical significance in the distribution of H3K9 di-methylation (Fig. 6A), H3K27 tri-methylation (Fig. 6B) and H3K4 tri-methylation (Fig. 6C) in the TNF-α promoter between normal and OA .SETDB1 (responsible for methylating H3K9), Ezh2 (responsible for methylating H3K27) and MLL2 (responsible for methylating H3K4) binding were also studied. The results showed no differences in SETDB1 (Fig. 6D), Ezh2 (Fig. 6E) and MLL2 (Fig. 6F) binding within the region in the TNF-α promoter between normal and OA patients.

## 4 Discussion

Inflammatory factors play important roles in OA, leading to the destruction and loss of articular tissues. As one of the most important players in inflammation, TNF-α and its production all play important roles in cartilage destructions. Results suggested that the TNF-α -G308A polymorphism can increase the risk of OA,^[20]^ Increasing the level of TNF-α in synovial fluid may accelerate the progression of OA,^[21]^ TNF-α and a disintegrin and metalloproteinase with thrombospondin motifs 4 are important in OA cartilage degradation.^[22]^ Moreover, Studied showed that TNF-α promotes the growth of breast cancer through the positive feedback loop of TNFR1/NF-κB (and/or p38)/p-STAT3/HBXIP/TNFR.^[23]^ In line with the above-mentioned reports about the association between TNF-α and OA, we collected tissue samples from human subjects to compare the TNF-α between OA knees and normal control and found that TNF-α is significantly higher as compared with the control. After confirmation of the association of TNF-α with risk of OA, the further focus is the molecular mechanism underlying the elevated production of TNF-α.

Epigenetics has been defined as heritable changes in gene expression that do not change the DNA sequence and include DNA methylation and histone modifications and microRNA expression.^[24]^ The loosening of the chromatin structure, exposure of the promoter region and the binding of transcriptional factors and RNA polymerase determined the gene expression of the mammalian,^[19]^ which is controlled by Histone modifications and DNA methylation. The results of this study revealed that the elevated production of TNF-α in subjects with OA could be attributed to the suppressed methylation of TNF-α promoter region. Thus, investigating the mechanisms underlying how epigenetic modifications regulate gene expression can provide deeper insight into gene expression regulation. Then next step for us is to explore the molecular mechanism underlying the hypo-methylation of TNF-α promoter.

We observed DNA hypo-methylation and histone hyper-acetylation in the TNF- promoter in OA cartilage compared with normal cartilage. We also found the binding of MeCP2, Dnmt3a and HDAC1 in the TNF-α promoter in OA cartilage compared with normal cartilage. DNA methylation, which catalyzed by DNA methyltransferase enzymes (DNMT1, DNMT3A and DNMT3B), plays a pivotal role in the regulation of gene expression. Methylation within the gene promoter regions lead to suppression of gene expression, whereas methylation within gene bodies just on the contrary.^[25]^ A study indicated that there are a wide variety of relationships between gene expression, DNA methylation and sequence variation in untransformed adult human fibroblasts.^[26]^ Many studies have shown that changes in the pattern of DNA methylation may be involved in the pathogenesis of OA. Methylation of DNA specific proteins binding to methylated CpG islands can result in stable and heritable transcriptional silencing. In our study, we also found that MeCP2 binding was reduced in the TNF-α promoter. This result indicated that MeCP2 binding may be dependent on CpG methylation within DNA sequence, and the function of MeCP2 was methylated CpG binding, more importantly, MeCP2 may also provide a binding platform to attract histone-modifying enzymes. It makes sense that DNA methylation, histone modifications, MeCP2 binding and histone-modifying enzymes binding as a whole, cooperated to control chromatin structure and gene transcription.

We studied the effect of histone deacetylation by studying histone deacetylases (HDACs). HDACs can provide histone tails which can promote high binding between the DNA backbone and histones, lead chromatin condensation and prevent transcription, then suppress gene expression. According to DNA sequence similarity and activities, the HDAC proteins are classified into two functional groups.^[27]^ Recent studies have shown that changing HDAC activity can lead to OA progression. Studies have shown that altered expression of HDAC4/HDAC7 can cause elevated expression of MMPS, suggesting that HDAC4/HDAC7 has a positive effect on the progression of OA.^[28,29]^

Histones methylation is an important modification resulting in the formation of active and inactive genomic regions which is associated with both transcriptional activation and silencing.^[30]^ Histone methylation adds one or more methyl groups to regulate transcription. Depending on the residue involve in the histones methylation, various transcriptional consequences occur such as the addition of three methyl groups to lysine 27 of histone 3 (H3K27) which results in transcriptional repression whereas methylation of lysine 4 in histone 3 (H3K4) leads to transcriptional activation^[31]^http://www.sciencedirect.com/science/article/pii/S1043661817309866?via%3Dihub-bib0380. But now, few reports are available regarding histone methylation in chondrocytes. We also found no differences in the status of H3K9 di-methylation and H3K27 tri-methylation in the TNF-α promoter region between normal and OA. This may suggest that H3K9 di-methylation and H3K27 tri-methylation may not be the main target to regulate TNF-α expression. A report showed that inhibition of histone acetylation by curcumin reduces alcohol-induced fetal cardiac apoptosis,^[32]^ and that histone deacetylases are target enzymes for cancer therapy,^[33]^ and some anti-methylation agents have been used in tumors treatments.^[34]^ We used gene engineering and exogenous inhibitors to alter epigenetic modifications and found that after these treatments, TNF-α overexpression in OA was suppressed by both. Although Dnmt3a overexpression and anacardic acid treatment may have other special effects, we can also prove that TNF-α promoter regions is sensitive to epigenetic treatments and that the overall effect on OA was the suppression of TNF-α over-expression. Previous studies also suggested a therapeutic effect of anacardic acid in the treatment of OA via regulating expression of IL-6 by modulating methylation status of the promoter region of IL-6.^[19]^ It is not a surprise to observe the therapeutic effect of one agent is mediated by its ability to modulate more than one signaling pathway such as IL-6^[19]^ and TNF-α as the case in this study. Therefore, anacardic acid could a potential therapeutic agent in the treatment of OA.

In this study, we investigated the interaction of the TNF-α between genetics, DNA methylation, histone acetylation and histone methylation and in the OA. Although the scope of epigenetics is very wide, many studies have not been carried out in-depth. The results suggested that the changes of epigenetic status regulate TNF-α expression in the cells, which are pivotal to the OA disease process.

The results showed that the methylation of the TNF-α promoter was promoted while the histone acetylation of TNF-α promoter was suppressed in OA cartilage, which led to the reduction of TNF-α expression. However, unlike methylation and histone acetylation, histone methylation was not significantly correlated with the pathogenesis of OA. These observations provide deeper insight into the pathophysiology of OA and can be used to develop new therapeutic methods to treat OA.

However, several limitations exist in our study. First, the forms of epigenetic modifications on TNF-α transcription are varied, including not only DNA methylation, histone acetylation and histone methylation, but also histone phosphorylation and redox modification.^[35,36]^ Since we only investigated the level of mentioned three canonical types regulating, further studies are needed to complete the puzzle of TNF-α epigenetic regulation pattern. Second, previous studies reported that the epigenetic modifications of TNF are correlated with patients’ age.^[37,38]^ But we didn’t adopt effective measures like case-control matching or stratified analysis to reduce biases caused by unstandardized demographic characteristics, which could impair the reliability of the conclusion.

In conclusion, we investigated the interaction of the TNF-α between genetics, DNA methylation, histone acetylation and histone methylation and in the OA. The results suggested that the changes of epigenetic status regulate TNF-α expression in the cells, which are pivotal to the OA disease process. These observations provide deeper insight into the pathophysiology of OA and can be used to develop new therapeutic methods to treat OA. However, further researches concerning the complete epigenetic modification mechanism of OA onset are still required to achieve greater progress.

## Author contributions

Conceptualization: Qiang Zhang, Xinqiao Tang.

Data curation: Zhengxiao Ouyang.

Formal analysis: Wei Zhu.

Investigation: Wei Zhu, Zhong Liu, Xiaoming Chen.

Methodology: Xiaoxia Song.

Resources: Zhong Liu.

Software: Zhengxiao Ouyang, Xiaoxia Song, Xiaoming Chen.

Writing – original draft: Qiang Zhang, Xiaoming Chen.

Writing – review & editing: Xinqiao Tang.
